# What Is IL-1 for? The Functions of Interleukin-1 Across Evolution

**DOI:** 10.3389/fimmu.2022.872155

**Published:** 2022-04-06

**Authors:** Diana Boraschi

**Affiliations:** ^1^ Shenzhen Institute of Advanced Technology (SIAT), Chinese Academy of Science (CAS), Shenzhen, China; ^2^ Institute of Biochemistry and Cell Biology (IBBC), National Research Council (CNR), Napoli, Italy; ^3^ Stazione Zoologica Anton Dohrn, Napoli, Italy

**Keywords:** interleukin-1, inflammation, innate immunity, adaptive immunity, evolution

## Abstract

Interleukin-1 is a cytokine with potent inflammatory and immune-amplifying effects, mainly produced by macrophages during defensive reactions. In mammals, IL-1 is a superfamily of eleven structurally similar proteins, all involved in inflammation or its control, which mainly act through binding to specific receptors on the plasma membrane of target cells. IL-1 receptors are also a family of ten structurally similar transmembrane proteins that assemble in heterocomplexes. In addition to their innate immune/inflammatory effects, the physiological role of IL-1 family cytokines seems to be linked to the development of adaptive immunity in vertebrates. We will discuss why IL-1 developed in vertebrates and what is its physiological role, as a basis for understanding when and how it can be involved in the initiation and establishment of pathologies.

## Introduction

After many studies that described soluble endogenous factors responsible for inflammation, fever and lymphocyte activation, human IL-1β was molecularly identified in 1984 as a single factor mainly produced by leukocytes (in particular monocytes and macrophages) responsible for many inflammation-related functions that occur during the host reaction to infections and other stressful events (1, 2). IL-1β is responsible for the induction of fever (originally described as “endogenous pyrogen”) (3–6), thereby contributing to create an unfavorable environment for infectious microorganisms and facilitate lymphocyte functions (which in mammals are more pronounced at temperatures above 37°C). Also, it amplifies B and T lymphocyte proliferation to antigens/mitogens (7–13), stimulates neutrophilia and acute phase proteins (14, 15), induces the production of proteolytic enzymes and prostaglandins by fibroblasts, chondrocytes and other cells (16–19), upregulates the expression and production of chemokines (IL-8, MCP-1) and inflammatory cytokines (TNFα, IL-6, IL-1 itself), induces the production of ROS and NO, causes redistribution of zinc and iron in the tissues and regulates corticosteroids and glucose homoeostasis (20–22), and is a potent adjuvant of antigen-specific antibody responses *in vivo* (23–29).

Thus, IL-1β appears to contribute to the defensive response by acting at different levels, *i.e.*, by directly participating to the inflammatory response (production of degrading enzymes and toxic molecules), by amplifying it (increase of body temperature, production of inflammatory factors, recruitment of effector cells, redistribution of nutrients and hormones) and eventually by non-specifically but directly activating/amplifying lymphocyte responses. The substantial disruption of homeostasis caused by IL-1β activation requires a complex and coordinated control system, which can inhibit IL-1 effects at various levels and dampen its activity once the defensive inflammatory reaction comes to an end. In addition, we should also consider that IL-1 is not a single molecule but rather a superfamily of structurally and functionally related cross-regulating cytokines.

A number of questions arise, when observing the plethora of IL-1 effects.

- Is IL-1 a typically innate/inflammatory molecule or is it involved in other functions beyond innate immunity? Why is it associated to vertebrates and to adaptive immunity? Does it have a physiological or homeostatic role? Does it have non-immune functions?
- Why do we have so many IL-1-like molecules that have overlapping effects? Is there a reason for such duplication of molecules, or is it just redundancy?
- Does IL-1 only act by binding to its receptors or does it have receptor-independent functions? Do the IL-1R have IL-1-independent functions?

In order to answer these questions, we need to know something more about the IL-1 superfamily.

### The IL-1 Superfamily

The cytokines of the IL-1 superfamily are proteins of about 17-18 kDa, with a typical β-trefoil pyramidal barrel structure composed by six two-stranded β hairpins, a structure common to many proteins with different functions throughout evolution, from bacteria to mammals, in particular for carbohydrate recognition (ricin-type lectins) and as toxins (30–34). In humans (and most mammals), eleven IL-1-like cytokines have been described, some with inflammatory activity and some with anti-inflammatory functions. From evolutionary analysis (see below), the ancestral IL-1 family encompasses IL-1β (the prototypical IL-1 cytokine) and other eight cytokines derived from it (IL-1α, IL-1Ra, IL-36Ra, IL-36α, IL-36β, IL-36γ, IL-37 and IL-38) (35, 36). IL-1β, IL-1α and the three IL-36 isoforms have largely overlapping inflammatory activities, with IL-1β and IL-1α binding to the same receptor (35, 36). However, IL-1α is a moonlighting protein, with a number of diverse functions attributed to its long intracellular form, which differ from the inflammatory functions of the extracellular shorter cytokine (37, 38). The three IL-36 isoforms (which all bind to the same receptor) have a range of tissue-specific inflammatory activities and are differentially present in different tissues (39, 40). Other members of the family have antagonistic or inhibitory activity: IL-1Ra is a receptor antagonist that binds in an inactive fashion to the same receptor as IL-1β and IL-1α, thereby antagonizing them (41); IL-36Ra is a receptor antagonist for the three IL-36 isoforms (34); IL-37 is an anti-inflammatory cytokine with a wide range of targets, possibly binding to the IL-18 receptor and to the inhibitory receptor IL-1R8 (35, 42–44); IL-38 was also reported to have anti-inflammatory activity (although its functions are not fully clarified) (45–47).

In addition to the ancestral family, the broader IL-1 superfamily also includes IL-18 and IL-33, which are not evolutionarily related, but have substantial structural and functional similarities with IL-1 (48). Both cytokines have multiple activities that are not strictly inflammatory and that are largely involved in immune metabolism. IL-18 is present in circulation at measurable levels (implying that it is not directly inflammatory), it induces the production of IFN-γ in T and NK cells in concert with IL-12, and it contributes to the differentiation of Th1 cells (49, 50). In its extracellular form, IL-33 is a potent activator of Th2 cells, as well as mast cells, basophils and eosinophils, thereby contributing to type 2 inflammation (51–53). In addition, intracellular IL-33 localises to the nucleus and presumably binds to DNA, although with unknown effects on gene expression, and it was reported able to interact with NFκB to decrease NFκB-dependent inflammatory gene transcription (51–53).

### The IL-1R Family

The receptors that bind the IL-1 superfamily cytokines are also structurally very similar, and are ten transmembrane proteins, which display an extracellular portion encompassing three Ig-like domains and a cytoplasmic portion that contains a Toll-IL-1R (TIR) signaling domain. The IL-1R complex that allows for cell activation is typically composed by a ligand-binding chain, which engages with the cytokine, and an accessory chain that associates with the dimeric ligand/receptor complex. The approximation of the two homologous intracellular TIR domains is the event that initiates signaling and eventual cell activation (54). The prototypical IL-1R is IL-1R1, the ligand-binding receptor for IL-1β and IL-1α. The accessory chain for IL-1R1 is IL-1R3, a promiscuous accessory chain that participates to several IL-1R complexes. Another ligand binding chain for IL-1β and IL-1α is the IL-1R2, which acts as a decoy receptor because it lacks the intracellular TIR domain and therefore can sequester ligands and IL-1R3 into an inactive receptor complex. The receptor antagonist IL-1Ra can inhibit binding of the active ligands IL-1β and IL-1α to IL-1R1. Other receptor complexes are similarly composed. The receptor for IL-36 encompasses the ligand binding chain IL-1R6 and the accessory chain IL-1R3, while the IL-33 receptor complex is composed by IL-1R4 and IL-1R3. The receptor complex for IL-18 includes the ligand binding chain IL-1R5 and a unique accessory protein, IL-1R7.

Other receptor-like chains included in the family have peculiar characteristics. The orphan receptors IL-1R9 and IL-1R10 are mainly expressed in the brain and do not have known ligands (54), although it has been proposed that IL-38 may bind IL-1R9 (46). The unique receptor IL-1R8 (also known as SIGIRR) is characterized by the presence of a single Ig-like domain in its extracellular portion and has the role of inhibiting receptor-mediated activation. Proposed mechanisms include interference with receptor complexes both at the extracellular and intracellular level (in this case active also in inhibiting TLR signaling), and acting as co-receptor for inhibitory ligands (hypothesized in the case of IL-37 bound to IL-1R5) (43). In teleost fish, another truncated receptor has been described, DIGIRR, which displays two Ig-like extracellular domains. DIGIRR is alternative to SIGIRR (same chromosomal location) and has similar anti-inflammatory capacity as SIGIRR, leading to hypothesize that it is a transition from between the classical receptors with 3 Ig domains and the single Ig domain SIGIRR (55).

A list of the human IL-1 and IL-1R molecules and genes, with some of the different names they are known with, is reported in **Table 1**, while **Table 2** summarizes the main known ligand-receptor interactions.

**Table 1:** Human IL-1 and IL-1R protein and gene names*.

IL-1 protein	Gene	IL-1R protein	Gene
IL-1β (IL1F2)	*IL1B*	IL-1R1	*IL1R1*
IL-1α (IL-1F1)	*IL1A*	IL-1R2	*IL1R2*
IL-1Ra (IL-1F3)	*IL1RN*	IL-1R3 (IL-1RAcP)	*IL1RAP*
IL-36α (IL-1F6)	*IL36A*	IL-1R4 (ST2)	*IL1RL1*
IL-36β (IL-1F8)	*IL36B*	IL-1R5 (IL-18Rα)	*IL18R1*
IL-36γ (IL-1F9)	*IL36G*	IL-1R6 (IL-36R)	*IL1RL2*
IL-36Ra (IL-1F5)	*IL36RN*	IL-1R7 (IL-18Rβ)	*IL18RAP*
IL-37 (IL-1F7)	*IL1F7*	IL-1R8 (SIGIRR)	*SIGIRR*
IL-38 (IL-1F10)	*IL1F10*	IL-1R9 (TIGIRR-2)	*IL1RAPL1*
IL-18 (IL-1F4)	*IL18*	IL-1R10 (TIGIRR)	*IL1RAPL2*
IL-33 (IL-1F11)	*IL33*	IL-18BP	*IL18BP*

*The names of the human proteins and genes are reported. Please note that while the gene names are unique, the proteins have been known with several names, the most common indicated in parentheses. The IL-18 binding protein IL-18BP is included in the list although not mentioned in the text and not belonging to the IL-1R family, because of its structural similarity with IL-1R proteins and its role in binding one of the IL-1 proteins.

**Table 2:** The IL-1 superfamily and the family of IL-1R*.

Ligand	Ligand-binding receptor	Accessory chain	Function	Notes
IL-1β	IL-1R1	IL-1R3	Activation	Inflammatory and immune-stimulating cytokine (Th17, CD8^+^)
	IL-1R2	IL-1R3	Lack of activation	
IL-1α	IL-1R1	IL-1R3	Activation	Moonlighting protein (different extracellular and intracellular roles)
	IL-1R2	IL-1R3	Lack of activation	
IL-1Ra	IL-1R1	IL-1R3	Inhibition of IL-1 binding/activation	IL-1 receptor antagonist
	(IL-1R2)	?	?	
IL-36α	IL-1R6	IL-1R3	Activation	Protective inflammatory cytokine
IL-36β	IL-1R6	IL-1R3	Activation	Protective inflammatory cytokine
IL-36γ	IL-1R6	IL-1R3	Activation	Protective inflammatory cytokine
IL-36Ra	IL-1R6	IL-1R3, IL-1R8?	Inhibition of IL-36 binding/activation	IL-36 receptor antagonist
IL-37	IL-1R5	IL-1R8?	Inhibition of inflammation	Inhibitor of inflammation
IL-38	IL-1R1?, IL-1R6, IL-1R9?	?	Activation? Inhibition of inflammation?	Both inflammatory and anti-inflammatory activities observed
IL-18	IL-1R5	IL-1R7	Activation	Protective inflammatory cytokine (Th1)
IL-33	IL-1R4	IL-1R3	Activation	Protective inflammatory cytokine (Th2), intracellular inhibitor of inflammation

*IL-1R10 is an orphan receptor with unknown function and therefore it does not appear in this table.

## Evolution of IL-1 and IL-1R

### Evolution of the IL-1 Superfamily

The evolutionary history of IL-1 and IL-1R offers very interesting implications and can help us understanding more about IL-1 functions. As mentioned above, the structure of IL-1 superfamily cytokines is quite common (β-trefoil barrel), as it can be found in molecules with different recognition and defensive functions across evolution, from bacteria and fungi (*e.g.*, pore-forming toxins and carbohydrate binding proteins) to plants (many lectins and toxins, Kunitz protease inhibitors), invertebrates (chitin-binding proteins and other lectins) and vertebrates (R-type lectins and domains in mannose and other receptors) (30–34). IL-1-like activities were extensively described in invertebrates (echinoderms, tunicates, insects) (56–66). However, no molecular characterization of invertebrate IL-1-like molecule(s) is at present available, and no gene orthologues were found in any clade besides vertebrates, based on the currently available genomes. This implies that IL-1 cytokines in vertebrates have taken functions that in invertebrates are performed by different molecules (58, 59, 63, 66). From evolutionary and chromosomal analysis, the ancestral proto-IL-1β gene appeared about 420 million years ago, *i.e.*, coincidental with the emergence of the vertebrate subphylum (48), encoding for a protein that adopted the β-trefoil barrel structure common to many invertebrate defensive molecules.

**Figure 1:**
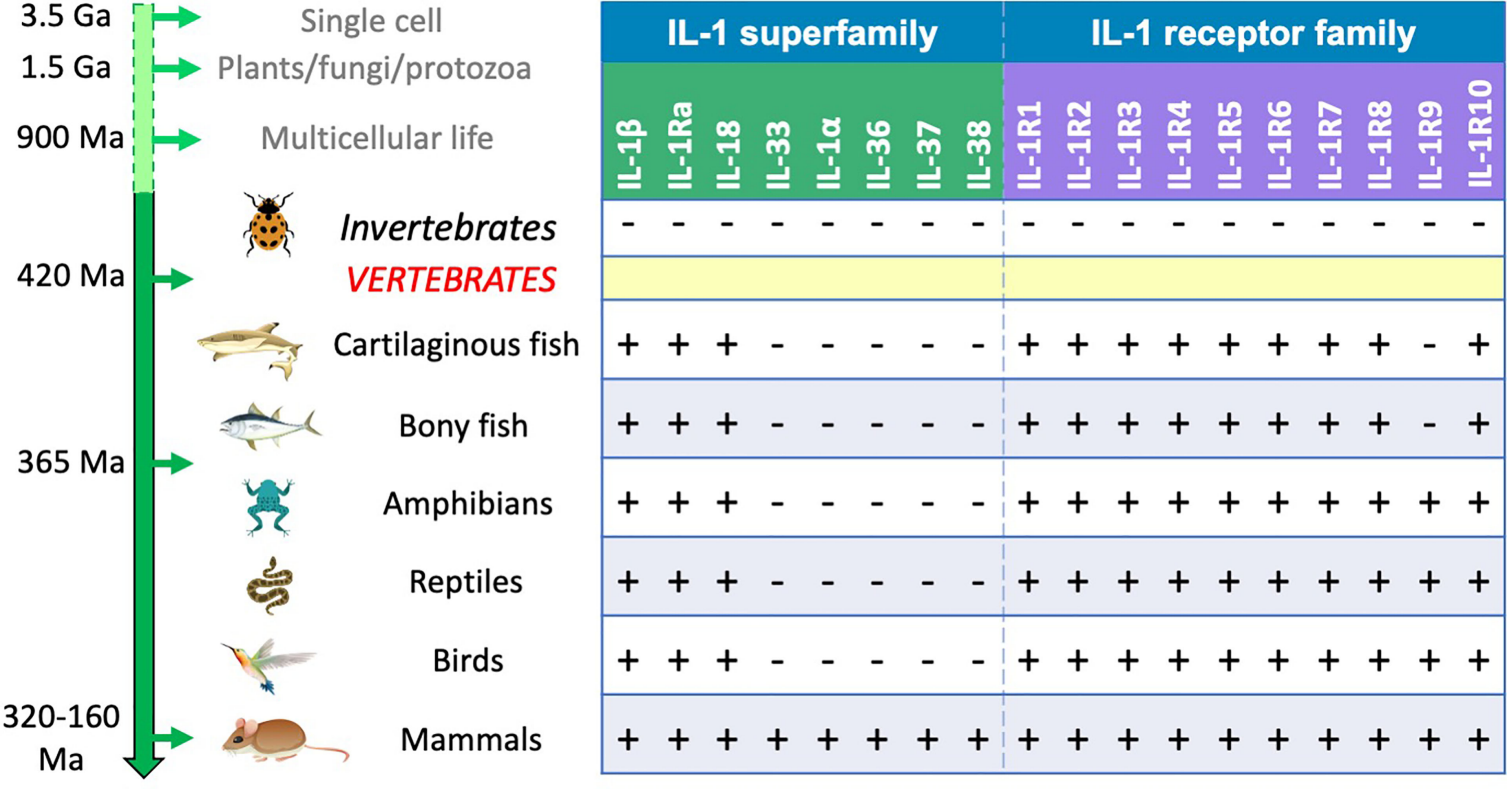
IL-1 and IL-1R family molecules in vertebrates. No IL-1-like homologous sequences have been identified in invertebrates, whereas numerous orthologues of IL-1R family genes were identified. This places the appearance of proto-IL-1 genes at the same time as the appearance of the vertebrate subphylum around 420 million years ago (48). The various IL-1R genes are present in all vertebrates, from cartilaginous fish to mammals, except for *IL-1RAPL1*, which only appeared after separation of bony fish and *Tetrapoda* clades about 365 million years ago, most likely from duplication of the *IL1RAPL2* gene. All vertebrates possess the genes for IL-1β, IL-1Ra and IL-18, whereas all the other IL-1 genes only appeared in mammals; genes encoding IL-1α, the IL-36 group, IL-37 and IL-38 are likely derived from IL-1β gene duplication, while the gene for IL-33 has independently evolved. Please note that the receptor genes were present in evolution long before the appearance of their ligands. Minus and plus signs indicate gene absence or presence.

From chromosomal location and surrounding gene positioning, three separate IL-1 families can be identified, *i.e.*, IL-1, IL-18 and IL-33 (48). While the IL-1 and IL-18 families are present in all vertebrate species, IL-33 only appeared in mammals. From the proto-IL-1β gene are derived all other cytokines of the ancestral IL-1 family, which are orthologues generated by gene duplication. Of them, only IL-1Ra is present in all vertebrates (independently evolved in fish, derived from proto-IL-1β gene duplication in other vertebrates), whereas IL-1α, the IL-36 group, IL-37 and IL-38 appeared much more recently (between 320 and 160 million years ago), as they are only present in mammals (48), similar to IL-33 (**Figure 1**). Why all the cytokines of the ancestral IL-1 family only appeared in mammals is a matter of speculation, although they have likely developed for performing specific defensive/immunostimulating functions linked to the peculiar characteristics of mammalian physiology and anatomy (large brain, fetal development, lactation, hair-covered skin, complex anatomy).

### Evolution of the IL-1R Family

IL-1R-like genes are present in invertebrates and are predicted to encode proteins with a typical organisation encompassing an extracellular Ig-like domain, a transmembrane region and an intracellular signalling domain. Several IL-1R-like orthologous genes, with homologies to the vertebrate genes, have been identified in *Cnidaria*, *Protostomia* and *Deuterostomia*, implying important yet unidentified functions (67–69). In addition, a significant homology can be observed between the intracellular portion of IL-1R and the Toll/Toll-like receptors (TLR), the most represented innate/developmental receptors in invertebrates, in particular in the signalling domain that has been therefore dubbed TIR (from Toll-IL-1R) (70, 71). Thus, IL-1R molecules are hybrid transmembrane receptors with the same signalling domain as TLR but with a distinct extracellular portion displaying Ig-like domains. While Ig domains are present in many invertebrate proteins, their inclusion into Ig-like receptors has expanded from pre-vertebrate chordata (68, 69, 72) to vertebrates giving rise to a plethora of receptors with high interaction capacity and specificity [including antigen-recognising T and B cell receptors; (73-75)]. In this perspective, the presence of Ig-like domains in the binding portion of IL-1R molecules is predictive of high binding specificity, at variance with the broad pattern-recognition capacity of TLR and other innate receptors.

The sequence similarity and the chromosomal anatomy of IL-1R genes in vertebrates suggest that most IL-1R molecules belong to a single family derived from duplication of an ancestral IL-1R gene. These are the two IL-1 receptor genes *IL1R1* and *IL1R2*, the IL-33 receptor gene *IL1RL1*, the IL-18 binding and accessory chain genes *IL18R1* and *IL18AP*, the IL-36 receptor gene *IL1RL2* and, by further duplication from one of the IL-1R genes, *IL1RAPL2* and (from it) *IL1RAPL1*, which is only present in birds and mammals (48). The only two IL-1R genes that do not seem to belong to the IL-1R family are the orphan receptor gene *SIGIRR* and the IL-1R3 gene *IL1RAP*, possibly derived from *IL18RAP* (48). *SIGIRR* shares low sequence homology and no chromosomal anatomy with the other genes, suggesting no common ancestry. As mentioned above, *SIGIRR* is likely the result of a gradual loss of extracellular Ig domains from a distinct IL-1R-like ancestral gene. IL-1R molecules are present in all vertebrates, except *IL1RAPL1*, which is absent in fish and only appeared in *Tetrapoda* about 365 million years ago (**Figure 1**). Given the presence of the intracellular TIR domain, the signalling pathways of IL-1R and TLR are similar, involving an overlapping range of downstream signalling and regulatory molecules, and leading to activation of overlapping immune defensive responses.

Notably, the appearance of receptors preceded that of ligands. This is obvious in invertebrates, in which IL-1R are present in the absence of IL-1-like molecules, but also in vertebrates, as in the case of IL-1R4 for IL-33 and IL-1R6 for the IL-36 group. The notion that receptors were present long before their cytokine ligands underlines their different original functions and their re-use in mammals for additional, IL-1-mediated effects. That IL-1R have appeared for functions that do not require IL-1 ligand binding is supported by the case of the orphan receptors IL-1R10 and IL-1R9, which do not have known ligands and that have non-immune functions in the brain (54).

## The Physiological Role of IL-1

### IL-1 Cytokines in Innate and Adaptive Immunity

Despite the common notion that IL-1 is an innate/inflammatory cytokine, the observation that IL-1 appeared in vertebrates, coincidental with the development of adaptive immunity, leads to hypothesize that its functions are not exclusively linked to innate immunity. The co-existence of innate and adaptive immunity in vertebrates likely led to a progressive “simplification” of the innate immune defensive mechanisms, which are particularly complex in several invertebrates (67). For instance, TLR and NLR genes decreased from over 200 in sea urchin to 10-20 in human beings. In this context, IL-1 family cytokines may have developed for taking a double role, to maintain a functional innate immunity that could also amplify the adaptive immune responses.

IL-1 family cytokines are considered innate immune factors because their production does not depend on cognate signalling and their effects are likewise non-specific. In the case of the prototypical cytokine IL-1β, its production is inducible, *i.e.*, the cytokine is produced upon stimulation, and there is no constitutive production in quiescent conditions, implying that its main role is in the reaction to triggering agents, typically infective microorganisms. Since the IL-1 receptors are receptors that signal through a TLR-like pathway, being TLR the main innate defensive receptors in invertebrate immunity, it is reasonable to say that IL-1β is a factor involved in immune defense, and we can therefore suggest that its main physiological role is inducing/participating to the innate immune protection from invasion and damage. However, the fact that IL-1 family cytokines are only present in vertebrates further suggests that their defensive activity goes beyond innate immunity and that they have a role in helping/amplifying adaptive immune responses.

IL-1β can non-specifically amplify T and B lymphocyte responses that, conversely, are triggered by specific antigenic signals. Thus, IL-1β can induce IL-2 secretion and expression of IL-2 receptors on T cells, induce expansion and commitment of naïve and memory CD4+ T cells (in particular Th17) in response to antigen and enhance their functions (76–87), thereby indirectly increasing B cell activation, and it can also increase CD8+ T cell activation and cytocidal capacity (88–90).

Likewise, the IL-1 superfamily member IL-18 is a potent activator of Th1 responses and participates to Th1 differentiation and activation, typically by inducing the production of IFN-γ in concert with IL-12 (49, 80, 91). IL-18 is constitutively produced by many cell types, and mainly released by monocytes and macrophages, and is present at measurable level in the circulation of healthy people, suggesting a homeostatic or surveillance role (91).

The other, most recent IL-1 superfamily member IL-33, present only in mammals, is an inducer of Th2 differentiation and amplification, thereby contributing to the specific defensive reaction against extracellular parasites (*e.g.*, helmints), bacteria and toxins (and to the overall type 2 inflammatory reaction) (51, 80), as well as to the successful establishment of pregnancy (92, 93).

### The Moonlighting IL-1 Superfamily Proteins

IL-1α is very similar in its effects as IL-1β in its mature extracellular form, including effects on T cell activation. This raises the question why, after duplication from IL-1β, IL-1α has divergently evolved and has been conserved in mammals. The most likely hypothesis is that IL-1α possesses different functions, non-redundant with those of IL-1β. Indeed, IL-1α is not a typical inflammatory cytokine, as its precursor protein (pro-IL-1α) is constitutively expressed in macrophages and many non-immune stromal cells (epithelial cells, endothelial cells, fibroblasts) in resting conditions, and it localises in the nucleus where it acts as transcription factor thereby regulating cellular functions, proliferation, senescence and apoptosis (37, 38, 94, 95). Upon inflammatory stimulation, pro-IL-1α can be cleaved and, while the C-terminal portion is exported outside the cell and acts as a cytokine (binding to the same receptor as IL-1β), the pro-piece can still act as transcription factor and upregulate the expression of inflammation-related genes (38, 95–98). Thus, the moonlighting nature of IL-1α, which has different functions in different conditions and in different compartments, is probably among the reasons why it has been conserved throughout mammalian evolution.

IL-33 shares with IL-1α its dual functions, both as a soluble IL-1-like cytokine active on Th2 cells and as an intracellular transcription factor constitutively present in several stromal cell types (37, 51). At variance with IL-1α, however, the nuclear IL-33 represses gene expression, as it does facilitate chromatin compaction (99), suggesting that IL-33 can act as a strong down-regulator of inflammatory responses and contribute to the re-establishment of tissue homeostasis during the resolution of a defensive reaction. Also, as mentioned above, the fact that IL-33 is only present in mammals suggests its major role in controlling inflammatory reactions during pregnancy, thereby facilitating embryo implantation and development (92, 93).

### Agonists, Antagonists and Inverse Agonists in the IL-1 Cytokine and Receptor Families

Based on the above notions, we may consider the IL-1 superfamily as a hybrid cytokine family, which has developed for amplifying and controlling adaptive immune responses in vertebrates but that is non-specific in its induction and effects, as it simultaneously displays typical innate/inflammation-related activities. The IL-1R characteristics contribute to the mixed activities of the IL-1 family cytokines, since the receptors display specific ligand recognition and binding (with their Ig-like extracellular domains) and are differentially expressed on the surface of distinct lymphocyte populations (thereby specifically targeting the IL-1 effects to them), but are typical innate receptors in their downstream TIR-dependent effects.

**Figure 2:**
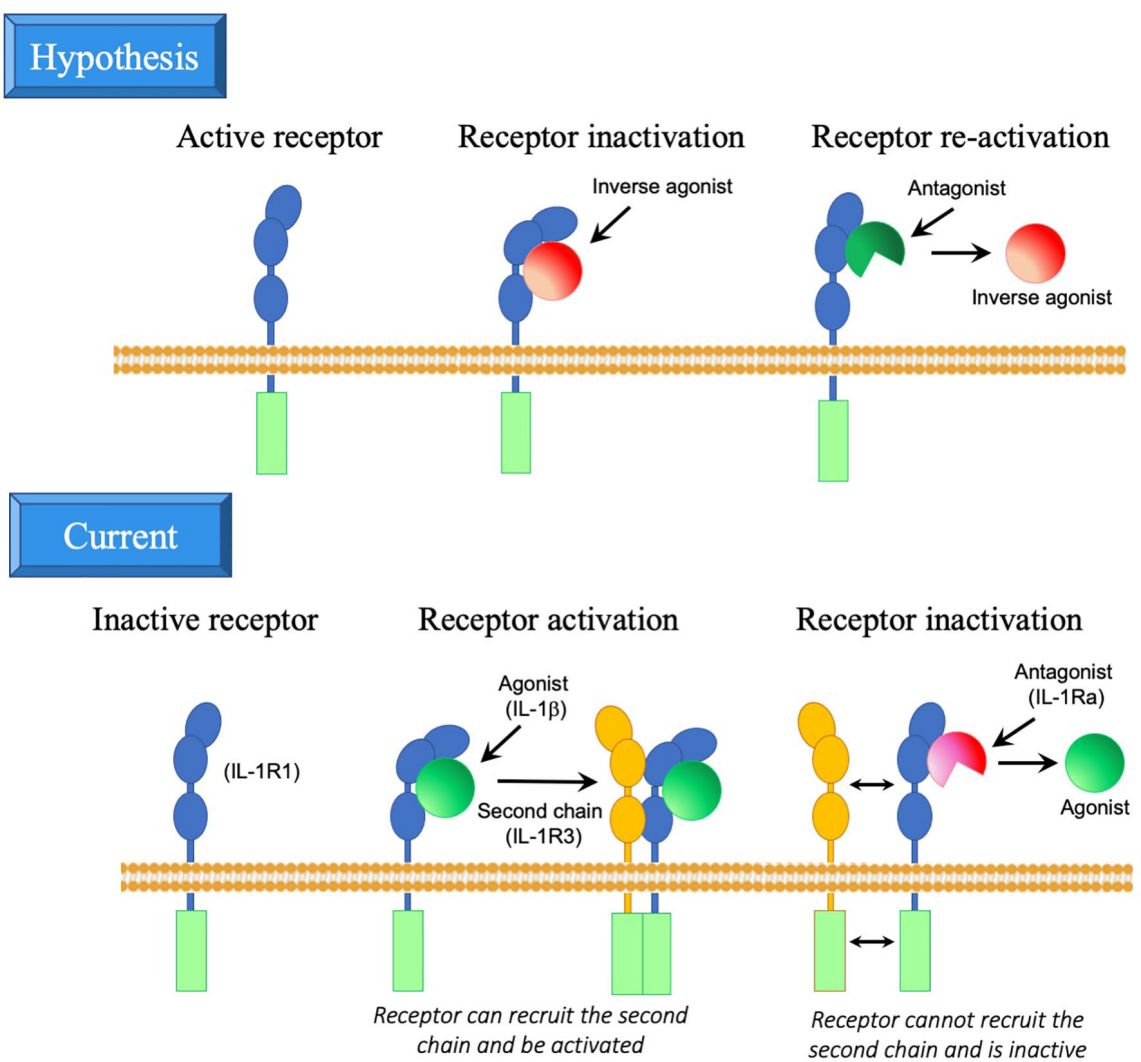
Inverse agonist hypothesis in the development of IL-1R ligands. Upper panel. Hypothesis on the possible development of IL-1R family molecules and their ligands. The receptor may have developed as a molecule with intrinsic activity, in the absence of interaction with ligands (this is still the case for instance for the orphan receptor IL-1R9). Ligands may have developed as a tool for inhibiting and modulating the receptor activity (inverse agonists, which inhibit rather than activate the receptor), likely by changing the receptor conformation. Consequently, antagonists may have developed as partial ligands, based on modifications of the inverse agonist structure, that bind to the receptor and displace the inverse agonists, while unable to modify the receptor conformation and its activity. Lower panel. Current model of IL-1R and ligand interaction. The receptor is intrinsically inactive (for instance, in the case of IL-1R1). Binding of an agonist (such as IL-1β) changes the receptor conformation thereby enabling the interaction with a second chain (such as IL-1R3) and consequent activation (by approximation of the intracellular domains of the two receptors). Antagonists (such as IL-1Ra) have a similar structure as agonists and are able to partially bind to the receptor, thereby displacing the agonist, but unable to modify the receptor conformation and thus preventing interaction with the second chain and consequent activation. *Artwork by W.J. Yang*.

However, the evolutionary data suggest a more complex scenario. Many of the IL-1R molecules appeared long before their IL-1 ligands, suggesting that these molecules had/have an original constitutive activity, independent of ligands. In mammals, this hypothesis is supported by some data showing ligand-independent receptor functions, as for instance in the case of IL-1R7 (100) and IL-1R9 (101, 102) in the brain. In the case of the IL-33 receptor IL-1R4, based on structural data, it has been proposed that its activation is brought about by interaction with the accessory chain IL-1R3 in a ligand-independent way, and that the function of IL-33 is merely that of stabilizing IL-1R4 in a conformation that promotes interaction with IL-1R3 (103, 104). We can hypothesize that the IL-1 ligands developed for downregulating and controlling the constitutive functions of such receptors, a strategy adopted by pathogenic microorganisms for inhibiting host receptors with antimicrobial capacity. These ligands would be inverse agonists, *i.e.*, agonists that dampen the activity of constitutively active receptors. Thus, the inverse agonist molecules may have appeared in evolution before the agonist ligands and may be derived from microbial genes/molecules (which, as already mentioned, share the same β-trefoil fold structure as IL-1 family molecules). In the case of microbial inverse agonists, the agonist ligands are the countermeasures created by the host organism to antagonise the microbial inverse agonists, thereby neutralising the pathogen’s escape strategy. Similarly, the IL-1 family agonists may have developed from the inverse agonists, based on modifications of the same structure, to re-activate, control and fine-tune biological functions that are down-regulated by an inverse agonist (**Figure 2**). IL-1Ra and IL-1β appeared around the same time, *i.e.*, about 420 million years ago with the emergence of vertebrates, underlining their mutual relationship, although we cannot know whether IL-1 emerged to counteract IL-1Ra or vice-versa. In mammals, IL-1R1 does not seem to have ligand-independent functions (which may have been lost during evolution), and IL-1β acts as agonist ligand, whereas IL-1Ra has mainly developed as antagonist ligand. However, it is notable that binding of IL-1β to IL-1R1 does not induce receptor activation, which can only be achieved after the engagement of a second non-ligand binding accessory chain. Thus, IL-1Ra does not counteract IL-1β by dumping IL-1β-induced receptor activation but rather by preventing interaction of the receptor with the accessory chain (**Figure 2**).

### Do IL-1 Family Cytokines Need Receptors?

IL-1 family molecules can exert a number of activities that are apparently independent of receptor engagement, as it has been reported in particular for the most ancient cytokines IL-1β and IL-1Ra. In ischemic brain damage, IL-1β can exacerbate damage also in the absence of IL-1R1, whereas IL-1Ra could only antagonise IL-1β in the presence of IL-1R1 (105). Likewise, IL-1β could modulate expression of a number of genes in primary microglial cells independently of IL-1R1, while other genes were only modulated depending on the receptor’s presence (106). In the same system, IL-1α only showed receptor-dependent effects (106). Most interestingly, IL-1Ra was observed having the same agonist activity as IL-1β in the hippocampus, with the IL-1Ra effects being IL-1R1-independent and those of IL-1β IL-1R1-dependent (107). The lack of correlation between receptor binding and activity in IL-1β is further supported by data on the IL-1R1-independent immunostimulatory capacity of the β-bulge surface loop between the 4^th^ and the 5^th^ β-strand in the IL-1β structure, and by the use of monoclonal antibodies and IL-1β mutants that showed a dissociation between IL-1R1 binding and different functional activities (108–112).

Notably, reminiscent of the lectin-like activity of β-trefoil proteins, IL-1β displays a lectin-like domain, distinct from the IL-1R1 binding sites, which can enable binding to specific carbohydrates (*e.g.*, heparin, hyaluronic acid, GM4), thereby promoting interaction with glycoproteins on cell membrane and in the extracellular matrix (113–118). This capacity of interacting with glycoproteins not only can promote the tissue-specific localisation of the cytokine, as initially proposed, but it can actually underlie a more complex and more controlled activation mode, entailing the concomitant or differential engagement of receptor and glycoproteins for activation/inhibition of immune functions (114, 115, 119). IL-1α also has a carbohydrate-binding domain, which however is different from that of IL-1β, implying a distinct interaction mode and distinct activity regulation (114, 115, 118, 120), while IL-1Ra does not have such domain (118).

All these observations support the hypothesis that IL-1β may have developed at first as a receptor-independent functional molecule, and that it has been subsequently “re-used” to counteract IL-1Ra effects, but still maintaining both receptor-dependent and independent functions within a complex interplay of regulatory cross-interactions.

## IL-1 Superfamily Cytokines in Pathology

The IL-1 family cytokine effects on immunity and inflammation imply that their dysregulation can cause pathological derangements. Excessive IL-1β is involved in a range of inflammation-related diseases, from chronic inflammatory diseases and autoinflammatory diseases to autoimmune syndromes, neurodegenerative diseases and cancer (2, 15, 35, 36, 41, 121). IL-1α released from necrotic cells is considered a major inducer of sterile inflammation in several ischemic conditions, by inducing neutrophil and macrophage recruitment and IL-1β expression and maturation (37, 121–123). The importance of IL-1β and IL-1α in sterile inflammation has been underlined by the clinical use of the recombinant IL-1Ra (Anakinra), which has allowed us to appreciate the involvement of these cytokines in many unexpected pathologies (41, 123), and by a rare genetic defect in IL-1Ra production that leads to a strong inflammatory pathology (124, 125). IL-1α in particular has also been linked to cancer development (95, 126, 127). Excessive IL-18 is associated to the development of autoimmune diseases, and the increased levels of IL-18 and of its inhibitor IL-18BP are a common marker of ongoing inflammation and ageing (49, 128–134). Increase in IL-36 is strongly linked to skin inflammatory diseases (39, 40, 134–136), while IL-33 can be involved in pathological type 2 inflammatory diseases, such as exacerbation of pulmonary inflammation in COPD and asthma/allergies (51, 134, 137, 138). Notably, the circulating levels of the soluble form of the IL-33 receptor IL-1R4 are also substantially increased in patients with inflammatory diseases, and high IL-1R4 levels are one of the predictive markers of heart failure (139).

Thus, all IL-1 cytokines, when dysregulated, can cause or participate to pathological outcomes, a notion that supports their role in maintaining immune functionality, *i.e.*, preparedness to mounting a defensive reaction and capacity to control it.

## Conclusions: Why Do We Have IL-1?

IL-1 family cytokines are a recent acquisition in the animal kingdom, suggesting that they are specifically needed for the optimal development and survival of vertebrates. IL-1 family cytokines have most likely emerged for improving the efficacy of the newly evolved adaptive immunity, which is highly specific (and therefore more efficient than innate immunity in reacting to individual dangers) but possibly too slow and too weak for an effective protection of the anatomically complex, multi-organ vertebrate organisms. Two different IL-1-like molecules independently appeared essentially at the same time as adaptive immunity, IL-1β and IL-18, implying distinct needs, most likely the amplification of strong anti-infective responses differentially initiated by Th17 and Th1 cells. IL-33 only appeared in mammals, although being involved in the activation of the more ancient type 2 responses. These responses are brought about by innate cells but also by Th2 cells (which are present in birds besides mammals). The need of IL-33 in mammals is therefore hypothetically linked to mammal-specific needs, such as pregnancy and embryonal implantation and survival, a process that implies the downregulation of type 1 responses (which would kill the embryo) and the enhancement of anti-inflammatory type 2 responses.

Thus, we can say that IL-1 family molecules are important for the adequate amplification of adaptive immune responses, acting by enhancing Th1, Th2, Th17 and CD8 functions. They act on adaptive immunity by using innate immune tools, through receptors that trigger innate immune responses and by using receptor-independent pathways that are still not fully known. Notably, the functions of IL-1 and IL-1R family molecules seem to be related to their organ localisation, with different molecules and different pathways and regulatory networks engaged in achieving tissue-specific immune protection. In addition, many questions are still open on the putative non-immune role of both the IL-1 family ligands and, importantly, their receptors, since the latter have evolved well before their ligands or do not have known ligands.

To the initial questions, we may try to give some answers, although still hypothetical.

- IL-1 family molecules indeed seem to have both homeostatic and inflammation-related defensive functions. Their presence in vertebrates underlines the association with adaptive immunity, and it seems that IL-1/IL-1R family molecules are important tools linking innate immune functions (lectin-like recognition domains in the ligands, TIR signalling domains in the receptors) with the amplification and regulation of adaptive immunity.
- The redundancy of IL-1 family cytokines is likely due to organ/tissue-specific activities and regulation, which may also explain why they have appeared in vertebrates (which have a more complex anatomy with specialised organs and organ-specific homoeostatic and defensive needs).
- That both IL-1 and IL-1R family molecules may retain ancestral non-immune functions or have developed them in specific organs (as the brain) is a realistic possibility, as it is also realistically possible that both IL-1 and IL-1R family molecules can have functions that do not depend on canonical ligand-receptor binding. The lectin-like interaction of some IL-1 cytokines with glycoproteins enlarges in a still unexplored fashion the range of their possible functions.

On these grounds, how IL-1 and IL-1R family molecules are involved in pathologies is still largely obscure, from a mechanistic point of view, and associations with diseases in most cases are made with circulating cytokine levels that may not reflect the relevant organ-specific alterations and may not imply a cause-effect relationship. Thus, our understanding of IL-1 family cytokines in health and disease is still at the beginning, and it will take a much deeper knowledge of their role in homeostasis and defence in different tissues and organs within the mammalian body before being able to harness their activity towards health promotion.

## Author Contributions

DB devised and wrote the paper. The author confirms being the sole contributor of this work and has approved it for publication.

## Funding

The author was supported by the EU Horizon 2020 projects PANDORA (GA 671881) and ENDONANO (GA 812661), the Italian MIUR InterOmics Flagship projects MEMORAT and MAME, and the Presidential International Fellowship Programme (PIFI) of the Chinese Academy of Science (2020VBA0028).

## Conflict of Interest

The author declares that the study was conducted in the absence of any commercial or financial relationships that could be construed as a potential conflict of interest.

## Publisher’s Note

All claims expressed in this article are solely those of the authors and do not necessarily represent those of their affiliated organizations, or those of the publisher, the editors and the reviewers. Any product that may be evaluated in this article, or claim that may be made by its manufacturer, is not guaranteed or endorsed by the publisher.
